# Sequencing of Pooled DNA Samples (Pool-Seq) Uncovers Complex Dynamics of Transposable Element Insertions in *Drosophila melanogaster*

**DOI:** 10.1371/journal.pgen.1002487

**Published:** 2012-01-26

**Authors:** Robert Kofler, Andrea J. Betancourt, Christian Schlötterer

**Affiliations:** Institut für Populationsgenetik, Vetmeduni Vienna, Wien, Austria; University of California Davis, United States of America

## Abstract

Transposable elements (TEs) are mobile genetic elements that parasitize genomes by semi-autonomously increasing their own copy number within the host genome. While TEs are important for genome evolution, appropriate methods for performing unbiased genome-wide surveys of TE variation in natural populations have been lacking. Here, we describe a novel and cost-effective approach for estimating population frequencies of TE insertions using paired-end Illumina reads from a pooled population sample. Importantly, the method treats insertions present in and absent from the reference genome identically, allowing unbiased TE population frequency estimates. We apply this method to data from a natural *Drosophila melanogaster* population from Portugal. Consistent with previous reports, we show that low recombining genomic regions harbor more TE insertions and maintain insertions at higher frequencies than do high recombining regions. We conservatively estimate that there are almost twice as many “novel” TE insertion sites as sites known from the reference sequence in our population sample (6,824 novel versus 3,639 reference sites, with on average a 31-fold coverage per insertion site). Different families of transposable elements show large differences in their insertion densities and population frequencies. Our analyses suggest that the history of TE activity significantly contributes to this pattern, with recently active families segregating at lower frequencies than those active in the more distant past. Finally, using our high-resolution TE abundance measurements, we identified 13 candidate positively selected TE insertions based on their high population frequencies and on low Tajima's *D* values in their neighborhoods.

## Author Summary

Transposable elements (TE's) are parasitic genetic elements that spread by replicating themselves within a host genome. Most organisms are burdened with transposable elements; in fact, up to 80% of some genomes can consist of TE–derived DNA. Here, we use new sequencing technology to examine variation in genomic TE composition within a population at a finer scale and in a more unbiased fashion than has been possible before. We study a Portuguese population of *D. melanogaster* and find a large number of TE insertions, most of which occur in few individuals. Our analysis confirms that TE insertions are subject to purifying selection that counteracts their spread, and it suggests that the genome records waves of past TE invasions, with recently active elements occurring at low population frequency. We also find indications that TE insertions may sometimes have beneficial effects.

## Introduction

Transposable elements (TE's) are mobile genetic elements that parasitize genomes by semi-autonomously increasing their own copy number within the host genome. This evolutionary strategy has been remarkably successful: most organisms harbor TE's, and they can constitute anywhere from 3–80% of genomic DNA [1]. TE insertions may sometimes confer an adaptive advantage to the host organism [2], [3], [4], [5], [6], even performing essential functions, as in the classic example of *Het-A* elements, which comprise the telomeric DNA of *Drosophila*. In this case, the transposition machinery is used to regenerate telomeric DNA lost during DNA replication [7], [8]. In most cases, however, TE's are a liability for the host organism. Active TE's are an major source of deleterious mutations [9], [10], [11]—in extreme cases, resulting in a syndrome of chromosome breakage and sterility called hybrid dysgenesis [12], [13]. Even in less extreme cases, TE insertions can disrupt the coding or regulatory sequence of genes, impairing their function [14], [15], [16], [17]. TE's may also impose more subtle costs, such as a metabolic cost on the host due to the translation of TE-encoded proteins, and the replication of genomic DNA laden with both inactive and active elements [18], [19]. And lastly, similar TE sequences inserted into non-homologous regions of the genome can induce ectopic recombination between these regions, resulting in deleterious chromosomal rearrangements and aneuploid gametes [20], [21], [22]. Quite recently, it has become apparent that transposition is repressed by a special class of small RNAs devoted to this purpose [23], [24].

Thus, the primary forces affecting the spread and maintenance of TE's in populations —transposition, countered mainly by repression of transposition and purifying selection— are understood in broad outline. But, even in *Drosophila*, where the study of the population dynamics and forces affecting the patterns of transposable element insertion densities and frequencies has a long history (*e.g,* [25], [26], [27]), the dynamics of transposable element evolution remain controversial. Two patterns, and their conflicting interpretations, are of particular interest. First, low recombining regions such as the pericentric heterochromatin or the tiny fourth chromosome are highly enriched for TE insertions [20], [22], [28], [29], [30], suggesting that selection against new insertions is weaker in these regions than in regions with normal recombination rates. This is unlikely to be entirely caused by a general reduction in the efficacy of selection in low recombining regions due to Hill-Robertson effects [22], [31], [32], [33], as these effects only rarely lead to the fixation of TE insertions in non-recombining chromosomes [33]. Instead, the abundance of TE's in these regions is rather due to either a low rate of recombination yielding a low rate of ectopic recombination [21], [22], [33] or to the small fraction of functional DNA in these regions [15]. Second, insertions from the same TE family tend to segregate at similar population frequencies [34], [35]. This might be due to families experiencing bursts of activity, such that insertions from the same family tend to be approximately at the same age [18], [36], [37], [38], and thus also roughly at the same population frequency. Alternatively, families might differ in properties that determine their equilibrium population frequencies—in their transposition rate and in the strength of selection against individual insertions [34], [35]. In this scenario, high copy number elements are expected to experience high levels of purifying selection, due to an increased opportunity for ectopic recombination [34], [35]. Elements of these families will thus tend to segregate at low frequencies, but the family itself will be maintained by a high overall transposition rate.

Population level studies of different TE insertions provide the best opportunity for resolving these controversies, but these studies have been hampered by the lack of an unbiased and practicable method of characterizing the frequencies of at which TE insertions occur at individual insertion sites. In the past, unbiased estimates of TE insertion frequencies (except for those of small insertions which may be missed) have been obtained by *in situ* hybridization of DNA probes containing TE sequences to the polytene chromosomes of different individuals [20], [22], [39], [40], [41], [42], [43], [44], but this technique has limited resolution and finds only relatively complete insertions. More recent studies have used PCR to survey populations for known insertions (*i.e*, insertions identified from a reference genome) [34], [35], [45], [46], but these surveys are necessarily biased towards insertions with high population frequencies, as those insertions are most likely to be found in the reference genome. Methods to survey population frequencies of TE insertions in an unbiased fashion do exist [47], [48], [49], but genome-wide methods require separate sequencing of the genomes of multiple individuals, which is usually prohibitively expensive. Here, we use a novel and cost efficient approach to identify TE insertions, regardless of whether or not they occur in the reference genome. Using this method, we analyze TE insertion frequencies from a Portuguese population of *Drosophila melanogaster*. We find that this population harbors large numbers of TE insertions not present in the reference genome: a conservative estimate suggests that there are almost twice as many novel as known insertions. Using the frequency estimates from the Portuguese population, we investigate evidence for the different models of transposable element evolution outlined above.

## Results

### Identifying TE insertion sites

**Figure 1:**
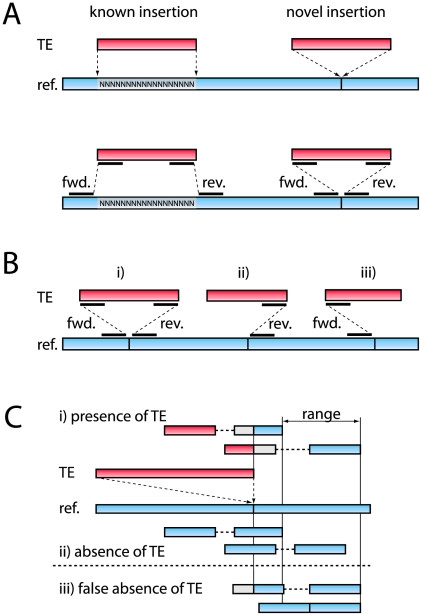
Outline of the method used to identify TE insertion polymorphism. (A) Top: Examples of a “known” insertion in the repeat masked reference genome, and of a “novel” insertion, not in the reference. Bottom: Paired ends mapped to known and novel insertions. (B) Three TE insertions identified by i) reads both 5′ and 3′ of the insertion site (forward and reverse insertions), ii) reads 3′ of the insertion site (reverse insertion), iii) reads 5′ of the insertion site (forward insertion). (C) Estimating the population frequency for a reverse insertion. First, the end positions of the reads confirming the presence of an insertion are recorded, and based on this information a range is defined. Subsequently, all PE fragments within this range that either confirm the absence or the presence of a TE insertion are tallied (see text). The reference genome and reads mapping to the reference genome are shown in blue. TE's and reads mapping to TE's are shown in red. Sequences not aligned by the Smith-Waterman algorithm are shown in gray.

We developed a method of identifying TE insertion sites, regardless of whether the insertion sites are known (present in the reference genome) or novel (*not* present in the reference genome). This method further provides estimates of the population frequencies of TE insertions without the large ascertainment bias that comes from sampling only TE insertions occurring in the reference genome. The method has three requirements: *(i)* an assembled reference genome *(ii)* a database of TE sequences, and *(iii)* paired-end (PE) sequences generated from the DNA of pooled individuals. The paired-end reads are mapped to a specially prepared reference genome, which consists of a repeat masked genome and the TE sequences used for repeat masking. A TE insertion is identified if one read of a PE fragment maps to a unique region of a reference chromosome and the other read maps to a TE (Figure 1A). We classified individual TE insertions using a nested hierarchy constructed from the information provided by FlyBase [50], with three primary orders (using the classification suggested by [1]) at the top level— one order of DNA-based elements, the terminal inverted repeat (TIR) elements, and two orders of RNA retrotransposons, the long-terminal repeat (LTR) elements and non-LTR elements. Within these orders, insertions are further classified into 115 families and 5,222 individual insertions (see Dataset S1). The use of a nested hierarchy allows us to operate at different hierarchical levels (mostly at the family level) thus facilitating identification of elements in spite of sequence divergence between the individual insertions (see Material and Methods).

Using this method, we characterized TE insertions in a *D. melanogaster* population from northern Portugal (Povoa de Varzim). To this end, we sequenced a sample of 113 isofemale lines and found that 11.4% of the aligned reads map to TE sequences, very similar to the proportion of sequences matching TE sequences (11.1%–13%) reported in a different study of a North American *D. melanogaster* population using low-coverage 454 shotgun sequencing [51]. In total, we identified 10,208 individual TE insertions (Table 1). These elements represent a broad taxonomic range, including 3,479 TIR insertions, 3,487 LTR insertions, and 2,975 non-LTR insertions (Dataset S2). To estimate the frequency of TE presence *vs.* absence at each insertion site, we first discarded low coverage sites (those having fewer than 10 reads) and overlapping TE insertions, as frequency estimates for these insertion sites are not reliable. We estimated the population frequency for the remaining 7,843 TE insertions (Table 1) as the ratio of the number of PE fragments showing the presence of the TE to total number of reads covering the site (Figure 1C; see Materials and Methods). As insertions present in the reference genome (“known” insertions) are expected to systematically differ in frequency from those that are not present (“novel” insertions), it is important that the method treats the two kinds of insertions equally.

**Table 1:** Abundance of TE insertions in the chromosomes of *D. melanogaster*.

chr.	length (Mb)		n^a^	density (#/Mb)	n_fe_ b	n_fixed_ c	fixed^d^ (%)
genome	120.4	all	10,208	84.8	7,843	2,702	34.5
		known	3,384 (5,222)	28.1	2,959	2,459	83.1
X	22.4	all	1638	73.1	1,315	402	30.6
		known	532 (856)	23.7	475	388	81.7
2L	23.0	all	1942	84.4	1,443	498	34.5
		known	587 (879)	25.5	521	424	81.4
2R	21.1	all	2099	99.3	1,596	693	43.4
		known	852 (1,323)	40.2	741	630	85.0
3L	24.5	all	2105	85.8	1,496	517	34.5
		known	628 (1,029)	25.6	528	444	84.1
3R	27.9	all	1938	69.4	1,604	255	15.9
		known	369 (590)	13.2	324	241	74.4
4	1.4	all	486	359.5	389	337	86.6
		known	416 (545)	307.7	370	332	89.7

a Number of identified TE insertions; All known TE insertions are in parenthesis.

b Frequency estimates where obtained for non overlapping insertions having more than ten absence or presence fragments.

c Number of fixed TE insertions (>0.95 frequency).

d Fraction of fixed TE insertions n_fixed_/n_fe_.

The total number of identified TE insertions (all) and the number of TE insertions present in the reference genome that have been identified by our approach (known) are shown. The numbers in brackets indicate the total number of TE insertions present in the reference genome. chr.: chromosome arm.

We assessed the reliability of our method in three ways. First, we asked how well we were able to identify known insertion sites. We identify 3,384 of the 5,222 TE insertions that are present in the reference genome (Table 1), suggesting that we may have missed a large fraction of reference insertions. However, not all of the reference insertions necessarily occur in any given population. Using our data (see Material and Methods), we estimate that 3,639 (69.7%) of insertions present in the reference genome also occur in the Portuguese sample, (very similar to the estimate of 69.4% present in samples from an African and a North American population in another study [51]). We suspect that the remaining 255 (7% of the 3,639 reference TE insertions) missed by our approach were either nested within other TE insertions (as overlapping insertions can be difficult to detect), or segregating at low population frequency and so missed by our survey (see below).

Second, we assessed the reproducibility of our population frequency estimates using the 2,035 insertion sites identified by reads at both sides of the insertion site. Reads on either side of an insertion site represent independent assessments of the TE population frequency (Figure 1A), and, reassuringly, the resulting frequency estimates were highly correlated for insertions detected from both directions (Spearman's rank correlation, *r_S_* = 0.902, *p*<2.2e-16). From the empirical distribution of population TE insertion frequencies, we estimate that we cannot reliably detect TE insertions below a frequency of about 7% in this data set (see Text S1), causing a slight overestimate of average population frequencies. This effect can be reduced by increasing the depth of sequencing coverage of the population, though nested insertions will remain challenging to detect. Furthermore, this bias is small compared to the one introduced by estimating only the population frequency for inserts found in the reference sequence. In fact, the fraction of insertions fixed in the Portuguese population (those with a frequency >95% in our sample) is very different for the reference insertions than for the sample as a whole (83.1% for reference insertions *vs.* 34.5% for all insertions; Table 1; these proportions are similar across all TE orders).

Finally, we assessed how well our method was able to reproduce (and extend) two well-established results: 1) underrepresentation of TE's in functionally important regions, and 2) overrepresentation of TE's in low recombination regions [29], [30], [52], [53], [54]. Functional sequence is known to show a paucity of TE insertions [29], [30], [35], [45]. Here, we see a clear contrast between intergenic TE insertions and insertions into regions with annotated functions in both TE densities and population frequencies (Table 2). Insertions in exons, which are expected to have strong functional constraints, are both rarer and segregate at lower frequencies than those in intergenic regions (Table 2), suggesting that exonic insertions experience significant negative selection. Dividing exons into coding sequence (CDS), 3′-UTRs and 5′-UTRs reveals that all three categories show a deficit of insertions and fixed insertions compared to intergenic regions, though not to the same degree. Not surprisingly, the evidence for negative selection is strongest for coding sequence. We further found that 3′-UTRs consistently contain more TE insertions than 5′-UTRs (Table 2), which has been reported before [45], [55], [56]. This may indicate lower density of functional elements in 3′-UTRs [45], and that insertions in these regions have fewer or weaker deleterious effects than in the 5′-UTRs. Alternatively, TE insertions in 3′-UTRs may provide some important function, such as polyadenylation signals [56], and may therefore be beneficial. We did not, however, find a significantly higher fraction of fixed TE insertions in 3′-UTRs as compared to 5′-UTRs (Fisher's exact test; TIR: *p* = 0.61; LTR: *p* = 0.20; non-LTR *p* = 1), suggesting that the difference between 5′-UTRs vs. 3′-UTRs TE insertions is not due to positive selection. Insertions in introns are also underrepresented (Table 2; see also [29], [30], [45],but not [35]), possibly due to disruption of regulatory sequences. This finding should be treated with some caution, however, as the inexact positioning of insertion sites may cause us to misannotate some exonic and intronic insertions. While we expect very little contamination of the intronic insertions, as exonic insertions are rare, we cannot exclude them.

**Table 2:** Abundance of TE insertions in different features of the *D. melanogaster* genome.

	feature	length (Mb)	n^a^	density (#/Mb)	n_fe_ b	n_fixed_ c	fixed^d^ (%)	median frequency
TIR	genome	120.4	3,479	28.9-	2,765	1,893	68.5-	1.00-
	intergenic	43.1	1,750	40.6-	1,364	984	72.1-	1.00-
	intron	47.0	1,617	37.6***	1,301	867	66.6**	1.00**
	exon	30.2	107	3.5***	96	38	39.6***	0.848***
	CDS	22.5	25	1.1***	22	4	18.2***	0.207***
	5′-UTR	3.5	14	3.9***	14	6	42.9*	0.531
	3′-UTR	4.8	66	13.7***	58	28	48.3***	0.948
LTR	genome	120.4	3,487	29.0-	2,569	388	15.1-	0.125-
	intergenic	43.1	1,726	40.1-	1,242	256	20.6-	0.143-
	intron	47.0	1,474	34.2***	1,132	115	10.2***	0.111***
	exon	30.2	286	9.5***	194	17	8.8***	0.100***
	CDS	22.5	169	7.5***	104	10	9.6**	0.088***
	5′-UTR	3.5	33	9.3***	28	4	14.3	0.106
	3′-UTR	4.8	89	18.5***	64	3	4.7***	0.120*
non-LTR	genome	120.4	2,975	24.7-	2,293	373	16.3-	0.122-
	intergenic	43.1	1,482	34.4-	1,119	223	19.9-	0.140-
	intron	47.0	1,372	31.9***	1,073	144	13.4***	0.111***
	exon	30.2	120	4.0***	100	6	6.0***	0.107**
	CDS	22.5	55	2.4***	42	2	4.8**	0.094***
	5′-UTR	3.5	21	5.9***	17	1	5.9	0.097
	3′-UTR	4.8	43	9.0***	40	3	7.5	0.150

a Number of TE insertions (including overlapping ones).

b Frequency estimates where obtained for non overlapping insertions having more than ten absence or presence fragments.

c Number of fixed TE insertions (>0.95 frequency).

d Fraction of fixed TE insertions n_fixed_/n_fe_.

- not tested.

***p<0.001.

**p<0.01.

*p<0.05.

The associated *p*-values indicate whether there is a significant difference from the intergenic regions, assessed by chi-square (for density) Fisher's Exact (for number of fixed insertion) or Mann-Whitney U (for median frequency) tests.

**Figure 2:**
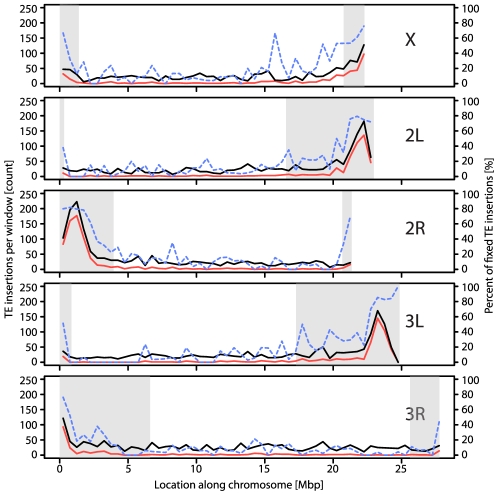
Distribution of all (black), fixed (red) and percentage of fixed (dashed blue) TE insertions in our natural population of *D. melanogaster*. The total number of TE insertions in a sliding window of 500 kb is plotted against the position in the five major chromosome arms of *D. melanogaster*. Shaded grey areas represent regions with a low recombination rate (<1 cM/Mbp).

Next, we examine our data set for expected excess of TE insertions found in low recombination environments. We find the highest density of TE insertions among the different chromosome arms on the low-recombining fourth chromosome (Table 1). Within each of the major chromosome arms, TE densities increase near the low recombining regions of the centromere proximal regions [22], [29], [30], [35], [39], [41], [46], [57] a result which we also find in our data set (Table 3; Figure 2; Dataset S3; Figure S1). As our method cannot reliably detect nested or clustered TE insertions, the enrichment of insertions near the centromeres is likely to be underestimated. In contrast to the low recombination regions near centromeres, we find no enrichment of TE's in the telomere proximal regions, in spite of their low recombination rates (Table 3; Figure 2; see also [29], [30]), with the exception of INE-1, a very old and abundant TE element (Table 3; [58], [59]) Note that the assemblies of the major chromosome arms used here do not include the subtelomeric heterochromatin, in which the domesticated *HeT-A* and *TART* elements reside [29]. We also found, as expected, that both the total number of insertions and the fraction of fixed TE insertions were strongly negatively correlated with recombination rate (with both density and recombination analyzed in 100 kb windows, excluding windows with <10 insertions; *r_S_* = −0.36; *p*<2.2e-16 and *r_S_* = −0.73; *p*<2.2e-16 respectively; see also [30], [35], [39], [41], [46], [57]).

**Table 3:** Abundance of TE insertions in telomere proximal, centromere proximal, and normal recombining regions of *D. melanogaster*.

		n^a^	density (#/Mb)	n_fe_ b	n_fixed_ c	fixed^d^ (%)
normal recombination	all	4,790	54.1-	3,985	399	10.0-
	TIR^a^	526	5.9-	430	46	10.7-
	INE-1	430	4.9-	366	267	73.0-
	LTR	1,968	22.2-	1,599	36	2.3-
	non-LTR	1,709	19.3-	1,465	36	2.5-
centromere proximal	all	4,547	178.8***	3,145	1,847	58.7***
	TIR^a^	596	23.4***	342	206	60.2***
	INE-1	1,371	53.9***	1,143	966	84.5
	LTR	1,374	54.0***	867	341	39.3***
	non-LTR	1,112	43.7***	718	305	42.5***
telomere proximal	all	385	75.5***	324	119	36.7***
	TIR^a^	31	6.1	22	8	36.4**
	INE-1	141	27.6***	123	101	82.1
	LTR	110	21.6	91	2	2.2
	non-LTR	91	17.8	76	7	9.2**

a Number of TE insertions (including overlapping ones).

b Frequency estimates where obtained for non overlapping insertions having more than ten absence or presence fragments.

c Number of fixed TE insertions (>0.95 frequency).

d Fraction of fixed TE insertions nfixed/nfe.

- not tested.

***p<0.001.

**p<0.01.

The recombination rate (<1 cm/Mbp) was used to delimit centromere proximal, normal recombining and telomere proximal regions. Note that order totals do not sum to overall totals since some TEs are not classified. The associated p-values indicate whether there is a significant difference to normal recombining regions.

### TE insertion frequencies in the Portuguese population

We estimate that, out of the 7,843 TE insertions for which we could obtain population frequency estimates, about one-third are fixed (34.5% at >95% population frequency), almost half are at low frequency (47.9% at <20% frequency), with the remainder segregating at intermediate frequencies (17.6% from 20 to 95%). That is, the distribution of insertion frequencies in the Portuguese population is U-shaped, with most insertions at either low or high frequencies. While TE insertions are well-represented in regions of normal recombination in our data set (just over half of the insertions with frequency estimates, or 3,985 of 7,843), most of the fixed insertions are in low recombination regions (83.1%, or 1,966 of 2,365).

Given that many TE insertions segregate at low frequencies [25], [35], [40], [41], [42], we might expect to find many insertions not present in the reference genome. In fact, this is the case: we detected 6,824 novel TE insertions, over twice the number of known insertions identified (3, 384 out of 5,222 present in the reference strain). These novel insertions have very different frequencies than those found in the reference sequence, with only 5% fixed, compared to 83.1% of the reference insertions. Consistent with the findings above, novel and known insertions tend to occur in different genomic regions. Most known insertions are in low recombination regions (78.8%, or 2,340 of 3,384; *χ^2^* = 5257.3, *p*<2.2e-16; see also [51]), and most novel insertions are in normal recombination regions (63.5% or 4,151 of 6,824; *χ^2^* = 566.5, *p*<2.2e-16).

**Figure 3:**
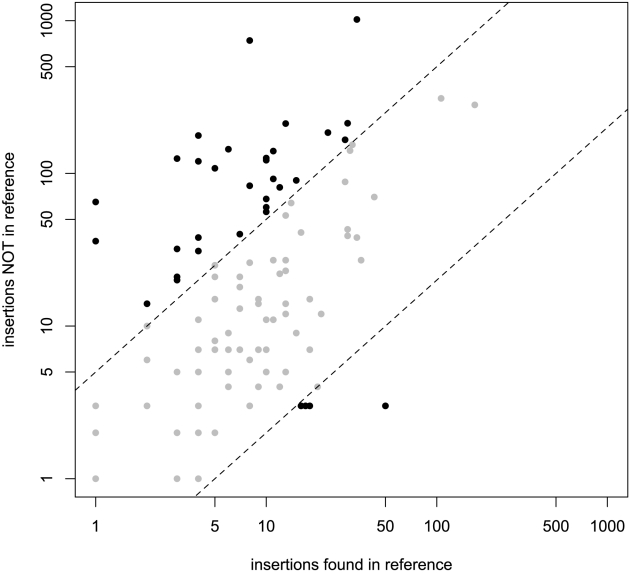
Number of novel identified TE insertions compared to known TE insertions for every TE family. Dashed lines mark the regions of five-fold difference between the number of novel and known TE insertions.

**Figure 4:**
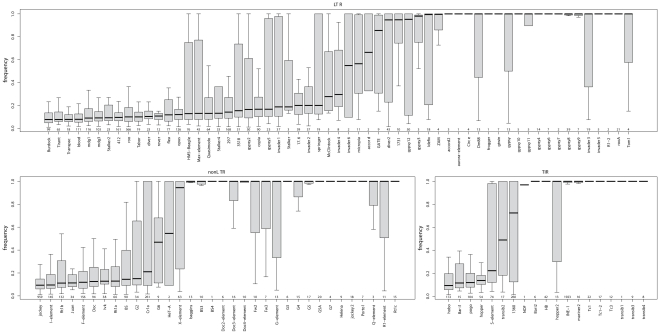
Boxplots of population frequencies in our natural population of *D. melanogaster* for all major TE families found in the Portuguese *D. melanogaster* population. The number of TE insertions whose frequencies are represented is indicated below the boxplot; only non-overlapping insertions were used to calculate population frequencies.

The relative fraction of novel to known insertions is highly variable among orders and families (Figure 3). That is, families differ significantly in the typical population frequencies of their individual insertions (Figure 4; effect of family: Kruskal-Wallis *χ^2^* = 4398.21, *p*<2.2e-16). Further, the families of the three TE orders differ in their median frequencies (Kruskal-Wallis *χ^2^* = 6.122, *p* = 0.043, p-values obtained by permutation), probably due to the abundance of low-frequency elements in the LTR order (Figure 4).

### Role of ectopic recombination in regulating population frequencies

Purifying selection has a strong potential to affect the density and population frequencies of TE insertions, as only TE insertions that do not disrupt important functions are free to drift to high frequencies. We confirmed above that TE insertions in functional sequence are rarer and, when they do occur, they have lower population frequencies than those in intergenic regions. But, in this data set, it is apparent that purifying selection on functional sequence cannot be the only evolutionary force affecting TE abundance. When we control for insertion into functional sequence, significant heterogeneity among insertions remains. Among intergenic insertions, there is still heterogeneity in population frequencies due to TE order and family [Kruskal-Wallis tests: family, *χ^2^* = 2125.8, *p*<2.2e-16; order (using median family frequencies), *χ^2^* = 10.013, *p*<0.002, *p* values obtained from permutation], suggesting that some property associated with these factors, such as its transposition rate or age (see below), affects insertion frequencies.

Importantly, the enrichment of TE insertions in low recombination regions does not appear to be solely attributable to a lack of functional sequence in these regions. That is, as low recombination regions have low gene density (the median number of exonic base-pairs per 100 kb window, excluding TE sequence, is 21,106 for low recombination regions and 25,569.5 for other regions; Mann-Whitney U-test, *U* = 168272, *p* = 2.4e-06), the argument could be made that the enrichment of TE insertions in these regions is due to the fact that TE insertions have fewer deleterious effects there [60]. But, this alone does not appear to explain the abundance of TE insertions in low recombination regions. Again restricting our analysis to intergenic insertions, we find that recombination still has a strong effect on TE density (number of insertions per 100 kb, *r_S_* = −0.311, *p*<2.2e-16) and on the population frequencies of insertions (*r_S_* = −0.504, *p*<2.2e-16). There is no evidence that this is because intergenic TE insertions are closer to genes in gene-dense high recombination regions, and therefore more constrained, as distance to the nearest gene has no effect on population frequencies of intergenic insertions (*r_S_* = −0.0005, *p* = 0.980). Moreover, even among exonic insertions, recombination plays a role in determining their population frequency (*r_S_* = −0.131, *p* = 0.008).

As the results above suggest that the concentration of TE insertions in regions of low recombination is not solely due to the lack of functional sequence there, we examined the role of recombination. It is generally assumed that the rate of meiotic recombination is positively correlated with the rate of ectopic recombination, although the exact relationship between meiotic recombination and ectopic recombination still needs to be determined. As ectopic recombination results in deleterious chromosomal rearrangements [21], [22], [61], it can cause purifying selection on insertions regardless of whether or not it disrupts functional sequence. We investigated the effect of recombination on polymorphic TE insertions. For this analysis, we excluded fixed TE insertions, the INE-1 family, and insertions on the fourth chromosome, as these insertions are potentially very old and thus unlikely to reflect ongoing purifying selection (see also [35]), and used only insertions in intergenic regions, to exclude the effect of purifying selection due to deleterious effects on genes. The remaining data set comprises frequency estimates for TE presence at 2,116 insertion sites.

We first confirmed that recombination rate also affects the frequency of these polymorphic elements, in addition to the effects on insertion density and fraction of fixed elements shown above. Insertions in low recombination regions are at higher frequencies than those in high recombination regions (*r_S_* = −0.101; *p*<0.0001; a negative correlation was found for all three orders, though the relationship is not significant for LTR elements; *p* = 0.059). We then examined our data for secondary factors affecting the rate of ectopic recombination. In addition to recombination rate, ectopic recombination between insertions is thought to be promoted by TE length, sequence similarity to other insertions, and the number of insertions from the same family [34], [35]. That is, insertions with more opportunity for mispairing —those with more extensive homology to paralogous sequence— should suffer correspondingly more from the effects of ectopic recombination than insertions with little similarity to other sequences. Some differences between TE families due to length could, instead, be caused by differential regulation of the piRNA pathway [23], [62], but stronger repression of transposition of a family should affect only the densities of new insertions, not their population frequencies.

We cannot explicitly explore sequence similarity or individual element length for most of the insertions in this data set, as we cannot recover sequence for insertions not present in the reference genome. However, we examine the effect of sequence length in two ways. First, we examine the effect of canonical length of a TE family on the median frequency of insertions from that family. We find a negative relationship between canonical family length and median family frequency (genome-wide, *r_S_* = −0.347, *p* = 0.0009; excluding regions of low recombination, *r_S_* = −0.064, *p* = 0.013). The interpretation of this result is complicated by the fact that the three major orders differ in both their typical lengths and in their median family frequencies (present study, [29]), but the relationship essentially holds within orders (though it is non-significant for the TIR order, which has very few families; Spearman rank correlation between median family frequency and length, LTR: 48 families, *r_S_* = −0.287, *p* = 0.048, non-LTR: 28 families, *r_S_* = −0.381, *p* = 0.050, TIR: 12 families, *r_S_* = −0.322, *p* = 0.308). Second, for the 123 polymorphic insertions that do occur in the reference sequence, we examine the effect of the length of individual insertions on population frequencies, and find that it is well-correlated with frequencies (*r_S_* = −0.33; *p* = 0.0002), even when only the 51 insertions in regions of normal recombination are considered (*r_S_* = −0.49; *p* = 0.0003; see also [35]).

As stated above, we expect TE insertions from families with large number of insertions to suffer more from the effects of ectopic recombination than those from families with few insertions. Consistent with this idea, we find a negative relationship between the size of a family (number of insertions genome-wide) and population frequency (*r_S_* = −0.178; *p* = <2.2e-16). There appears to be no additional effect of local element density, as a higher number of insertions from the same family in the immediate vicinity (within 1 MB) does not reduce population frequencies (*i.e*, there was no significant negative correlation between frequency and local density for any of the families with more than 30 insertions, *r_S_* = −0.153–0.362, *p* = 0.009–0.993), suggesting that ectopic recombination may not occur more often between nearby sequences.

Interestingly, the *X* chromosome does not have the lowest density of transposable elements, as might be expected given its overall higher rate of recombination ([22]; insertions per Mb for *X* = 73.1, *2L* = 84.4, *2R* = 99.3, *3L* = 85.8, *3R* = 69.4, *4* = 359.5; Dataset S4) or from direct effects of TE insertions in hemizygous males of *D. melanogaster* [20] (see also [63], [64]). However, these numbers cannot be compared directly, as chromosomes differ in the extent of low-recombining heterochromatin. Controlling for recombination rate, using an analysis of covariance, and ignoring the fourth chromosome, we find that there is no effect of *X*-linkage on the number of TE insertions per MB window (ANCOVA on ranks: median rank adjusted for *X*-linkage = 57.9; autosomal = 59.3; *F*_1,120_ = 3.53; *p* = 0.063; recombination rates *not* adjusted for *X*-linkage in this analysis only).

Finally, and perhaps surprisingly, we find that there is no correlation between recombination rate and frequency when we exclude regions of low recombination from the analysis (*r_S_* = −0.028, *p* = 0.274), suggesting that a small amount of recombination is sufficient to exert the whole effect of recombination on population frequencies. Accordingly, it might be the case that ectopic recombination is not directly related to recombination rates measured in genetic crosses, or that forces other than ectopic recombination are more important influences on population frequencies of TE insertions in the euchromatin.

To obtain an overall picture of the factors affecting TE frequencies, we used linear models to examine these factors in details, an approach previously used by [35], [37]. We examine several factors that should affect the rate of ectopic recombination: canonical family length, the recombination rate (adjusted for X-linkage in the standard way), the number of polymorphic insertions from the same family, and the number of polymorphic insertions of the same family in the neighborhood of the insert (within 1 MB). We also explore factors which might influence population frequencies by means other than ectopic recombination, such as chromosomal arm and distance to the nearest gene. Note that because of correlation between some explanatory variables in the model, we cannot use the model to make inferences about their strength of the effects (that is, the regression coefficients are not reliable); however, the overall fit of the model is valid [65], and is what will be assessed here.

We fit a linear model to the log-transformed frequencies, using the covariates listed above and their second order interactions. The insertion family and chromosome are added as factors. As we cannot simultaneously examine both family and order, we include only the family in the model, after confirming that using family is preferable to using order (Table 4). We then iteratively added terms to our model and used AIC as criterion for retaining the terms in the model. Consistent with the results above, the canonical length, number of polymorphic insertions in a family, recombination rate, and family are retained in the model (Table 4). Interestingly, the number of insertions in a MB region and the distance to the nearest gene, which had no significant effect above, are also retained, although dropping the distance to the nearest gene and its interactions had a minimal effect on the AIC (without distance to gene: AIC = −1110.1; Table S1). Not surprisingly, given the result above, dropping recombination rate as a continuous covariate and replacing it with a factor denoting whether the insertion is in a region of normal recombination, in the telomere proximal regions or in the centromere proximal regions improves the fit of the model (Table 4), again suggesting that a small amount of recombination is sufficient to reduce population frequencies of TE insertions. However, this picture may change with future improvements in estimates of the recombination rate, or a richer understanding of how homologous and ectopic recombination rates are related, particularly for telomeric regions [22], [66].

**Table 4:** Models fit to TE polymorphism data.

	Polymorphic TE insertions				polymorphic insertions (large families only)	
	Full model, using family	Full model, using order	Reduced model (using rec rate)	Reduced model (using TEC)	Reduced model (using rec rate)	Reduced model (using TEC)
Canonical length	+	+	+	+	+	+
Distance to nearest gene	+	+	+	+	+	+
Global family density (polymorphic insertions in family)	+	+	+	+	+	+
Local family density (polymorphic insertions in family within 1 MB)	+	+	+	+	+	+
Recombination	+	+	+	+	+	+
Taxonomy (family or order)	+	+	+	+		
Chromosome arm	+	+	+	+		
Canonical length*Distance to nearest gene	+	+	+	+		
Distance to nearest gene* global family density	+	+			+	+
Canonical length * local family density	+	+				
Canonical length * global family density	−	−	−	−	+	
Distance to nearest gene* local family density	+	+				
Global family density * local family density	+	+	+	+	+	
Canonical length * recombination	+	+	+	+		
Distance to nearest gene * recombination	+	+			+	+
Global family density * recombination	+	+	+	+	+	
Local family density * recombination	+	+				
Rank (age)	−	−	−	−	+	+
Rank (age) * local family density	−	−	−	−		+
Rank (age) * canonical length	−	−	−	−	+	+
n	2110	2110	2110	2110	671	671
Model d.f.	104	22	99	102	13	13
Model R-squared	0.209	0.108	0.208	0.215	0.136	0.1311
AIC	−1102.49	−1023.5	−1109.23	−1123.68	−389.8	−385.7

Models containing the full set of independent variables and their second order interactions were fit to log-transformed population frequencies of polymorphic TE insertions (full models). For the reduced models, we started with the full model containing family (rather than order) as a factor, and dropped or retained independent variables using AIC as the criteria (reduced models), with either the recombination rate or the centromere proximal, normal recombining or telomere proximal regions (TEC) used to indicate recombination environment. In a separate analysis, we fit models to the subset of data from the 11 families with age estimates and more than 30 insertions. We started with all the terms in the full model (with order as the taxonomic level), as well as the rank age estimates and second order interactions with age. The terms in the models are indicated by ‘+’ in the table; terms not tested are indicated by ‘−’.

### Role of TE family age

The patterns of population frequencies of insertions detailed above are attributable to ectopic recombination, but also have an alternative interpretation under a different model of TE evolution. That is, if TE families may have bursts of activity [18], [36], [37], [38] followed by long periods of inactivity, the different histories of different TE families will affect the characteristics of insertions examined here. Recently active families should show a large number of insertions segregating at low frequency, while recently inactive families should have fewer insertions, as many insertions will have been lost, while the remaining insertions are mostly fixed. For example insertions from the *INE-1* family, which has not been active for >3 million years [58], [59], are mostly fixed (Figure 4). (Interestingly, we found that not all insertions of the *INE-1* family are fixed (82% fixed; >0.95 population frequency). This could be due to either to a false absence reads leading to biased estimates of the population frequency (see Text S1), or, alternatively it is possible that not all insertions of *INE-1* are fixed species-wide. In fact we find 124 *INE-1* insertions that are not present in the reference sequence (45% of which are fixed in our sample), showing that at least some of these insertions are not completely fixed, as otherwise they would be present in the reference genome). Moreover, recent insertions will, on average, be longer than old insertions, as the well-documented deletion bias in *Drosophila* [67], [68], [69] will have had less opportunity to reduce the size of these insertions. In other words, the associations between frequency, length and number of TE insertions (although not the relationship with recombination rate) found above might be a consequence of recent activity of a family.

Assuming the burst model of family activity outlined above, we use the median age of 27 TE families (as estimated [37]) and ask how this affects population frequencies. We found that the estimated age of a family was well-correlated with its average population frequency (*r_S_* = 0.802; *p* = 4.72e-07; Dataset S5), suggesting that time of activity of a TE family has a strong impact on the population frequency spectra. There is a potentially confounding effect, however, as the age estimates of [37] are based on levels of sequence diversity in a TE family within the *D. melanogaster* genome. If sequence diversity affects the rate of ectopic recombination, such that insertions with many mutations enjoy a lower rate of ectopic recombination than those with very few, purifying selection alone might result in a negative correlation between diversity and frequency. To address this concern, we repeated the analysis, by using TE families thought to have recently invaded the *D. melanogaster* genome through horizontal gene transfer [70]. We find that these families show a significant enrichment of low population frequency insertions (Wilcox Rank Sum test; *W* = 234; p = 0.0042; Dataset S6).

To assess whether including the estimated age of a family improves the predictions of population frequencies, we also separately analyzed the subset of 794 insertions from the 11 element families for which we have age estimates and large numbers of insertions, allowing us to compare the effect of age and recombination rate. Because we are interested in comparing these family level effects, we drop family as a term in the model, but add order, and used age rank of the family to avoid any dependency on the specific age estimates. Using the add-drop procedure, age is retained in the model, regardless of the proxy used for recombination rate (Table 4). Removing age substantially worsens the model (with adjusted recombination rate: AIC = −380.1, with telomere proximal, normal recombination, and centromere proximal as proxies for recombination rate: AIC = −361.7; Table S1).

### Identification of putative positively selected TE insertions

As our sample shows substantial number of polymorphic TE insertions, they may provide considerable material for adaptive evolution. In fact, among the few well-documented cases where we know the target of adaptive evolution at the genetic level in *Drosophila*, at least two are due to transposable element insertions. In one case, insecticide resistance is conferred by an *accord* insertion close to the *Cyp6g1* gene, which results in increased expression levels of that gene [4]. In the other example, also involving insecticide resistance, a *Doc* insertion into the *CHKov1* gene disrupts the gene, yielding an alternative set of transcripts [3].

We thus investigate the possibility that some of the insertions fixed in the Portuguese population were fixed *via* positive selection. We again ignored *INE-1* insertions in this analysis, as *INE-1* has not been active for >3 million years [58], [59], and thus are unlikely to be the targets of recent positive selection. We considered TE insertions in fixed in high recombining regions as promising candidates for recent positive selection, as fixed insertions in regions of normal recombination are unusual (132 cases, or <3% insertions when low recombination regions are excluded). To distinguish selection from genetic drift, we also required candidates for positive selection occur near regions of low Tajima's *D* values (*TD*) [71]. Negative values of *TD* indicate an excess of rare mutations, one possible signature of a sweep due to positive selection [72]. We identified insertions in the neighborhood of *TD* lower than the genome wide 5% quantile (*i.e*, within, or immediately adjacent to, windows of 500 bp with TD<−2.265 for the autosomes and TD<−2.397 for the X chromosome). We included the flanking windows of the actual TE insertion in this analysis, as the strongest signal of selection may not be directly at the site under positive selection [73].

As a result of this analysis, we identified 13 putatively positively selected TE insertions (Table 5), of which 5 are located on the X chromosome and 8 on the autosomes. We asked if two TE insertions involved in the adaptation to insecticides are among the candidates, an *accord* insertion close to the *Cyp6g1* gene [4] (not in the reference genome), and a *Doc* insertion into the *CHKov1* gene [3] (insertion FBti0019430 in the reference genome). Both were among the candidates identified here (Table 5). We also compared our results to a different set of putatively positively selection TE insertions identified by [5], and found that only the *Doc* insertion mentioned above overlaps between the two studies. This is likely due to the different criteria used— fixation in a single population (present study) *vs.* a frequency difference between African and non-African populations [5].

**Table 5:** Candidate positively selected TE insertions.

nr.	chr.	pos.	Family	order	sup.	freq	TE ID	−2	−1	0	+1	+2	closest gene	location	putative function
1	X	3,680,043	mdg1	LTR	F	1.00	FBti0019564	−1.5282	**−2.4234**	na	−2.2869	−2.2165	FBgn0086899	intron	regulation of cell shape
2	X	4,582,532	HMS-Beagle	LTR	FR	1.00	FBti0060479	−2.4301	−1.9550	−2.2732	**−2.4301**	−2.0763	FBgn0011760	intron	actin filament bundle assembly
3	X	17,000,405	Ninja-Dsim.	ninja	FR	1.00	FBti0062283	−1.1942	**−2.4992**	na	−1.5800	−2.3464	FBgn0065032	520 bp us	actin filament organization
4	X	18,678,871	Rt1b	non-LTR	FR	0.98	FBti0019082	−2.0867	**−2.5178**	na	−1.7790	−1.9961	FBgn0030958	987 bp us	actin binding
5	X	20,254,231	3S18	LTR	R	1.00	FBti0019655	−2.2056	**−2.4012**	−2.2527	**−2.5641**	−2.0739	FBgn0085340	380 bp ds	unknown
6	2L	13,783,837	S-element	TIR	R	1.00	FBti0060388	−2.2020	**−2.3683**	na	−2.0966	−2.3344	FBgn0028539	252 bp us	transporter activity
7	2R	5,758,108	rooA	LTR	R	1.00	FBti0061742	−0.4752	**−2.3306**	−0.8749	**−2.3972**	−1.771	FBgn0011241	intron	spermatoid development
8	2R	8,072,887	Accord	LTR	F	1.00	-	−2.3306	**−2.2964**	−1.7256	−2.2527	−2.0249	FBgn0033693	3′-UTR	unknown
9	2R	11,540,143	Roo	LTR	R	1.00	-	−1.8110	−2.0345	**−2.2919**	−1.6543	−0.6354	FBgn0260429	2328 bp ds	unknown
10	2R	13,919,899	Hobo	TIR	F	1.00	FBti0059793	−1.7469	**−2.3530**	−0.4077	−1.6845	−1.9715	FBgn0034289	9988 bp ds	unknown
11	3L	12,181,292	gypsy12	LTR	R	1.00	FBti0063191	−0.8079	**−2.3796**	−0.5754	−1.8479	−1.9908	FBgn0036262	332 bp ds	oxidation-reduction process
12	3R	7,394,212	G5	non-LTR	F	1.00	FBti0020329	−1.5106	−1.7627	−1.9876	**−2.4011**	−1.7754	FBgn0025701	760 bp us	wing disc dorsal/ventral pattern formation
13	3R	21,152,377	Doc	non-LTR	F	1.00	FBti0019430	−2.0306	−1.6583	na	**−2.2963**	−1.9980	FBgn0045761	CDS	RNA-dependent DNA replication

Tajima's *D* values below a threshold value (see Material and Methods) are indicated in bold. Sup. indicates whether support for the TE insertion comes from forward reads (F), from reverse reads (R) or from forward and reverse reads (FR); freq.: frequency of the TE at the insertion site; −2, −1, 0, +1, +2: Tajima's *D* values for nonoverlapping windows of 500 bp surrounding the TE insertion. The window containing the TE insertion has a offset of 0, windows 5′ of the TE insertion have a negative offset (−1, −2) and windows 3′ of the TE insertion have a positive offset (+1, +2). us: upstream; ds: downstream.

Of the 13 insertions identified as putatively positively selected here, 11 are present in the current annotation of *D. melanogaster* (5.31). These candidates belong to very different TE families and orders, with 2 TIR insertions, 8 LTR insertions and 3 non-LTR insertions. The location of these insertions with respect to the nearest gene varies: 4 are upstream of the next gene, one is in the CDS, 3 are in introns, one is in the 3′-UTR, and 4 are downstream of the next gene. The putative functions of these genes nearest to candidate TE insertions are diverse, ranging from wing disc pattern formation to spermatoid development (Table 5), and show no significant enrichment for a gene ontology category. The fact that only 2 of these insertions are in exons suggests that positively selected TE insertions mostly have an influence on expression of genes by *cis*-regulatory effects. The 3 intronic insertions may instead yield alternative transcripts.

These insertions represent only candidates for positive selection, and we cannot exclude other possibilities. For example, it may be that the TE insertion happens to be near the target of a selective sweep, resulting in a low *TD*, while the causative mutation is elsewhere. This may have been the case for the candidate TE insertion close to the *Est-6* gene (FBti0063191), which was identified as ancestral, predating the split of *D. melanogaster* and *D. simulans* [51]. It has further been suggested that the *cis*-regulatory region of the *Est-6*, which co-segregates with the TE insertion has been the target of positive selection [51], [74].

Alternatively the regions neighboring the sweep may have exceptionally low *TD* for stochastic reasons, such as fluctuations in population size [75]. Furthermore, low TD values may partly be caused by non-synonymous sites, as we found that windows with low TD values contain exons slightly more often than other windows (low TD: 39.7%, high TD: 31.7%; Fisher's exact test; p<2.2e-16). We also examined the regions near our candidate insertions for a depression in nucleotide variability (see Figure S2) expected near strong selective sweeps [76]. We note, however, that such a signature strongly depends on the history of the selection event. Only for selective sweeps starting from a low population frequency is a pronounced trough in variability expected around the causative mutation. Given this limitation, we consider the fact that nine out of 13 candidates show a visually recognizable trough in variability a strong support for the non-neutral history of these TE insertions.

The true number of positively selected TE insertions in our natural population of *D. melanogaster* may, for several reasons, be higher than the 13 candidates presented in this work. That is, in addition to ignoring insertions in low recombining regions and nested insertions, our criteria also exclude incomplete sweeps, and may sometimes exclude sweeps fixed from standing variation [77], [78], which have been shown to contribute to TE insertion mediated adaptation [3], [5]. Hence, the number of TEs that contribute to adaptation of natural *D. melanogaster* populations may be substantially larger than this estimate indicates.

## Discussion

In this work, we have developed a method for the identification of population frequencies of TE insertions in pooled populations using paired-end sequencing (Figure 1). The primary advantage of this method is that it does not require previous knowledge of TE insertions allowing for relatively unbiased estimates of their frequencies. This is a substantial improvement over some prior methods, which measure TE polymorphism only at insertion sites known from the reference genome (e.g, [79]) and suffer the attendant ascertainment bias problems [35], [79]. Sequencing and assembling of every individual separately also allows ascertainment bias free frequency estimates, but is costly and error prone, as repetitive regions are notoriously difficult to assemble [80]. In contrast, our method requires sequencing of one pooled sample for the population of interest, a reference sequence and an appropriate TE database. Extension to other species with sequenced genomes should thus be straightforward. However, it does suffer a few limitations. It cannot identify insertions from TE families not in the supplied TE database, identify clustered and nested TE insertions, distinguish full-length from partial insertions and the locations of insertions are only roughly estimated. And finally, insertions segregating at low population frequencies can be missed, depending on the depth of coverage.

Because the method treats novel and known insertions equally, we are able to estimate the frequencies of large numbers of insertions whether or not they are present in the reference genome. In fact, most (66.8%) of the insertions identified in the Portuguese population were *not* present in the reference genome. The abundance of non-reference insertions is a natural consequence of the population frequency distribution of insertions, which tend to be either rare or fixed in this data set, consistent with previous reports based on smaller data sets [34], [35], [40], [42], [45], [46], [57], [81]. That is, the high proportion of TE insertions segregating at low frequencies— *e.g*, 48% occur at frequencies lower than 20%— implies that there will be many TE insertions not captured in the reference genome. Sampling more individual lines and/or increasing the coverage of insertion sites (which averages 31-fold in our study) will increase the number and proportion of novel insertions, as more rare insertions will be sampled. A similar effect can be seen in our data set by lowering the number of paired-end fragments required to identify and insertion from three to two, in which case the fraction of novel insertions increases to 81% and the number of novel insertions more than doubles (from 6,824 to 14,786).

Our improved frequency estimates confirm many inferences from previous work, but provide a more complete picture of the evolutionary forces acting on insertions within populations and put individual observations into context. For example, the population frequency of TE insertions strongly depends on the sampled TE families and orders (Figure 4; [35], [51])—*e.g*, DNA transposons were more frequently fixed than RNA transposons— and the sampled regions (Figure 2). Thus with different sampling strategies vastly different estimates of TE population frequencies will be obtained, which could be an explanation for the conflicting reports about TE population frequencies. In particular, estimates of frequencies of insertions from *in situ* methods are mainly limited to the euchromatin, where most insertions segregate at low population frequencies (Figure 2; [25], [41], [42], [57]) and may have missed the fixation of many elements in heterochromatic regions for technical reasons [46]. In contrast, when frequencies of insertions in the reference sequence are examined, most TE insertions are in low recombination regions, and are fixed or at appreciable frequencies (*e.g*, [35], [46], [51]).

Further, our data shed light on the nature of the forces affecting TE insertions in populations. These data provide evidence that ectopic recombination might counteracts the spread of TE insertions through populations, and that the abundance of TE insertions in low recombination regions is not, or not entirely, due to less functional selection in these regions. But they also suggest that an equilibrium model where transposition rate and purifying selection due to ectopic recombination are the primary factors affecting population frequencies may not provide a complete picture. New families invade the *Drosophila* genome [82], and recent successful invasions, coinciding with bursts of activity due to derepression, and the time since these bursts must have some influence on element frequencies. In fact, we see that the age of TE families, estimated from phylogenetic data, appears to be well correlated with population frequencies. And age may also contribute to the relationship between length and frequency—old, high frequency insertions will accumulate deletions and thus be short [46]. The strongest argument against age having an effect on element frequencies is that it requires a recent increase in activity in many LTR families to explain the abundance of low frequency/high copy number families in this order [35]. However, it is plausible that the enrichment of young TE families is due to rapid turnover in LTR families [36], [37]. That is, it may be that new LTR families invade *Drosophila* species more frequently (perhaps due to higher rates of horizontal transmission [38], [70] or differential targeting by small RNAs [38], [83]), and are also lost faster (due to more frequent ectopic recombination) than families from other orders.

Finally, our novel method of characterizing TE insertion population frequencies can be applied to any organism with a well-assembled reference genome. Application to other organisms will demonstrate the generality of the patterns observed in *Drosophila*.

## Materials And Methods

### Fly samples and sequencing

We sequenced 113 isofemale lines cultured from *D. melanogaster* females collected in 2008 from northern Portugal (Povoa de Varzim), as described previously (PoPoolation DB [84]). The lines were kept in the laboratory for five generations, and five females from each line were combined into a pool of flies for sequencing. DNA was extracted from homogenized female flies with the Qiagen DNeasy Blood and Tissue Kit (Qiagen, Hilden, Germany) for generation of paired-end libraries using the Genomic DNA Sample Preparation Kit (Illumina, San Diego, CA). Briefly, 5 µ*g* of DNA were sheared with a nebulizer and after end repair, A-tailing, and ligation of paired-end adapters, the library was size-selected on an agarose gel (300 bp) and amplified using 10 PCR cycles. Cluster amplification was performed using a Paired-End Cluster Generation Kit v2. Sequences were generated with the Illumina Sequencing Kit v3 and the Genome Analyzer IIx. Image analysis was performed with the Firecrest, Bustard and Gerald modules of the Illumina pipeline v. 1.4. In total, we sequenced 5 paired-end lanes, which produced 80 mio PE fragments (160 mio individual reads) with an average read length of 74 bp.

### Identification of TE insertion sites

The goal of the mapping procedure used here was to identify cases in which one read of a paired end fragment maps to a TE, and the other maps to a location in the *Drosophila* genome. To achieve this, we used the *D. melanogaster* reference genome v 5.31 and transposable element sequences obtained from FlyBase (http://flybase.org/; [29], [85]). We retained only TE sequence having a length greater or equal to 40 bp). We also masked repeat sequences in the reference genome using RepeatMasker open-3.2.9 [86] with the rmblast 1.2 search engine (parameters: -no_is -nolow -norna -pa 4) using the length filtered TE sequences form FlyBase as custom repeat library. We then constructed a combined reference sequence consisting of the repeat masked reference genome of *D. melanogaster* (v5.31) and the length filtered TE sequences. We then mapped our ∼160 million paired end reads to this combined reference sequence using BWA-SW 0.5.7 [87] with default settings. BWA-SW uses a Smith-Waterman algorithm [87], which allows for a partial mapping of the reads, potentially useful for reads spanning a TE insertion site. As BWA-SW does not support mapping of paired end reads, paired end information was recovered using a custom Perl script (samro). The mapping results were further processed using samtools 0.1.8 [88]. Both paired-end reads were mapped for 69.6 million (86%) out of the 80.5 million PE fragments (Table S2). We identified 961,283 PE fragments indicating the presence of TE insertions, i.e.: PE fragments with one read mapping to the reference chromosome and the other one to a TE. Unexpectedly, the number of PE fragments confirming a TE insertion from the forward direction (forward reads) and the number from the reverse direction (reverse reads; Figure 1A) were unequal (414,123 reverse reads; 547,160 forward reads; Fisher's Exact Test; p<2.2e-16). We can only speculate as to what causes this bias, with one possibility being the heuristics applied in the BWA SW algorithm [87].

We clustered forward and reverse reads into distinct TE insertion sites, limiting this analysis to TE insertion fragments in the non-heterochromatic reference chromosomes (2L, 2R, 3L, 3R, X, 4), using a two step protocol. First, we clustered reads in the same direction if they: *(i)* were separated by less than 225 bp (insert size+2×standard deviation the average distance between reads of a PE fragment) and *(ii)* mapped to the same TE type (*e.g*, INE-1). We further required that an insertion be supported by a minimum of 3 PE fragments, each with a minimum mapping quality of 15. We identified 6,672 insertions by forward reads, and 6,566 by reverse reads; note that this ratio of clustered forward and reverse reads is more balanced than that of the unclustered ones. Next, we combined adjacent forward and reverse insertions of the same family separated by between 74 and 250 bp intro single insertions (where the minimum distance is the read length, and the upper limit is empirically determined to result in the lowest levels of misclustering, see below). In order to treat TE insertions that are in and not in the reference genome equivalently, we ignored repeat masked sequence in the reference genome in calculating the distance between forward and reverse insertion sites. Using this procedure, we identified 10,076 individual TE insertion sites. Our procedure for clustering forward and reverse reads is based on distance, and so may therefore sometimes result in incorrectly grouping multiple TE insertions, or, conversely, erroneously splitting single TE insertions into two. We estimated the accuracy of the clustering procedure using TE insertions known from the FlyBase annotation (v5.31). We assumed that a TE insertion identified in our data corresponds to an insertion in the reference sequence if both insertions belong to the same family, and if the paired reads supporting the insertion map to within 300 bp of the reference insertion. This analysis showed that a total of 150 insertions were erroneously clustered together, while 18 were falsely split. For further analysis, we corrected the clustering for these TE insertions, resulting in a total of 10,208 TE insertion sites, with 3,030 identified by both forwards and reverse reads (n_2_) and 7,178 TE insertions solely by forward or solely by reverse reads (n_1_; Figure 1B).

### Estimating the number of reference TE insertions missed by our method

We estimated the total number of known TE insertions present in the sample using the following method. Let *p* be the probability of identifying a reference insertion present in the population, let *n_1_* be the number of reference TE insertion only identified by reverse or forward reads, and let *n_2_* be the number of reference TE insertions identified by both forward and reverse reads. Let *n_T_* further be the total number of reference insertions present in the sample. If the probability of identifying a reference insertion (*p*) is equal across insertion sites, then it is binomially distributed, with: *n_1_* = 2*p*(1−*p*)*n_T_*, and *n_2_* = *p_i_* ^2^ *n_T_*. Given the direct estimates of *n_2_* and of *n_1_* from the data (see above) *p* and *n_T_*. can be estimated. It follows that *n_0_*, the number of TE insertions *not* identified, can be calculated as: *n_0_ = n_T_−n_2_−n_1_* This analysis was conducted for each TE order separately.

### Estimating the frequency of TE occupancy at insertion sites

We estimate the frequency at which a TE is present at individual insertion sites as the ratio of the number of PE fragments that support the presence of the insertion (“presence fragments”) to the total number of reads covering the physical insertion site (including both “presence” and “absence” fragments; Figure 1C). While this is simple in principle, a practical difficulty arises from the fact that the precise TE insertion site is not known for all novel TEs, and, in these cases, we cannot determine with certainty whether a pair of reads map to either side of an insertion site, indicating the absence of the TE. Hence, we used the presence fragments to empirically define two 100 bp ranges in the reference genome on either side of the insertion site where we expect absence reads to map (Figure 1C: “range”). By truncating these ranges to 100 bp, we avoid overestimating the size of the ranges due to presence fragments with unrepresentatively large insert sizes, which could lead to an overestimate of the number of absence fragments. To estimate frequencies, we use only reads mapping within these ranges to tally either the presence or the absence of an insertion. Specifically, we considered absence fragments to be those where *(i)* both reads map in a proper pair, *i.e*, both reads map to the same reference, with the read mapped to the forward strand followed by the read mapped to the reverse strand, and with no overlap between the reads, and *(ii)* the end position of the 3′ read (or, for forward insertions, the start position of the 5′-read) maps within the 100 bp range (see Figure 1C for an example of a reverse insertion). We considered presence fragments to be those where *(i)* one read aligns to a TE sequence and the other read to the reference genome, and *(ii)* the position (end position for reverse reads and start position for forward reads) of the read mapping to the genome is within the same range as that used for the absence fragments. If a TE insertion is only identified by forward or reverse reads, the frequency estimate is solely based on the forward or reverse reads; otherwise, we averaged the estimates obtained from forward and reverse reads. We discarded insertion sites with lower than 10-fold coverage (defined as the sum presence and absence fragments), and TE insertion sites with overlapping ranges, yielding a total of 7,843 TE insertions with population frequency estimates (Table 1). See Text S1 for an assessment of the reproducibility of these frequency estimates.

### Statistical and population genetic analysis

Recombination rates for *D. melanogaster* were obtained for 100 kb windows from http://petrov.stanford.edu/cgi-bin/recombination-rates_updateR5.pl. The exact position of a TE insertion cannot be determined with our method, so we approximated positions using either the midpoint between forward and reverse reads identifying an insertion, or for TE insertions only identified by reads from one direction, using the last (first) position occupied by a forward (reverse) read plus (minus) 26 bp (1/3 inner distance between paired end reads).

We used the Flybase annotation to determine the functional category of the sequence surrounding the insertion, with categories expected to have stronger functional constraints taking precedence, as this is conservative for our purposes [in order of priority: exon (which can be further divided into CDS, 3′ UTR, 5′ UTR), ncRNA, regulatory, intron and intergenic]. We used chi-square tests to compare the number of TE insertions in a feature to the number in intergenic regions, and Fisher's exact test to compare the number of fixed and polymorphic TE insertions to those in intergenic regions. To analyse population frequencies, we used either the non-parametric Mann-Whitney U test, or linear models on log-transformed data. For linear modeling, we attempted to use arcsine transformed frequencies and generalized linear models with binomially distributed errors, but qq plots showed that these models fit poorly, while linear models fit to the log-transformed data fit well. As many of the tested models are non-nested, we used AIC to test model fit. Reduced models were obtained using the “step” function in R, which adds and drops terms based on AIC.

We calculated Tajima's *D* in non-overlapping 500 bp windows using PoPoolation v1.2.1 [89]. To do this, we trimmed (trim-fastq.pl, with base quality threshold of 18 and minimum length of 50) PE reads and subsequently mapped them to the *D. melanogaster* genome (5.31) using BWA 0.5.8 (parameters: -l 150 -n 0.01 -o 2 -e 12 -d 12). Paired-end information was restored using BWA SAMPE (0.5.8), and reads were filtered for unambiguous positions with samtools (0.1.8) [88] using a minimum mapping quality of 20. We converted the reads into a pileup file using samtools (0.1.8). The pileup file was sub-sampled to a uniform coverage of 30 bp using random sampling without replacement, a maximum coverage of 250 and a minimum base quality of 20. Tajima's *D* values were calculated using a minimum count of one and a window size of 500 bp; Tajima's π values were calculated using a minimum count of one and a window size of 2,500 bp.

For each of the candidate insertions, the nearest gene, the relative location with respect to the nearest gene and the ID of known TE insertions were obtained visually with IGV (1.5.06) [90], using the annotation of *D. melanogaster* (5.31). Putative functions of genes were obtained from FlyBase (http://flybase.org/) using either the first biological function, if available, or when not available the first molecular function. Analysis for an enrichment of GO terms was performed using FuncAssociate 2.0 [91].

### Software and data

The data are available from the European Nucleotide Archive (http://www.ebi.ac.uk/ena/) with the accession number SRA035392. The software used in this work is distributed as PoPoolation TE and available at Google Code (http://code.google.com/p/popoolationte/).

## Supporting Information

Figure S1 Distribution of TE insertions and their population frequencies in *D. melanogaster*. The x-axis shows the position in the chromosome and y-axis the population frequency of a TE insertion. Light grey insertions indicate low recombining regions (<1 cM/Mbp).
(PDF)

Figure S2 Nucleotide diversity in the vicinity of the 13 candidates of positively selected TE insertions. Nucleotide diversity was calculated for non-overlapping windows of size 2.5 kbp in a region of 100 kbp.
(PNG)

Dataset S1 The TE hierarchy used for this analysis.
(TXT)

Dataset S2 Population frequency estimates for all TE insertions.
(TXT)

Dataset S3 Distribution of TE insertions and fixed TE insertions along the major chromosomes of *D. melanogaster* using a sliding window approach with a window size of 500 kb. Data are shown for TIR, LTR and non-LTR insertions.
(XLS)

Dataset S4 The TE composition for the six major chromosome arms of *D.melanogaster*. The TE composition is provided for families and orders separately.
(XLS)

Dataset S5 Correlation of the age of TE insertions and the average population frequency.
(XLS)

Dataset S6 Population frequencies for TE insertions showing evidence for horizontal gene transfer.
(XLS)

Table S1 Models fit to TE polymorphism data.
(XLS)

Table S2 Mapping statistic for the 5 paired end lanes used in this study. Paired-end (PE) reads have been mapped to a combined reference consisting of the masked genome and the TEs of *D. melanogaster*. (chr: chromosome; TE: transposable element; fwd: forward; rev: reverse).
(XLS)

Text S1 Supplementary discussion.
(DOC)
