# Supplementary figures and images for: Sequencing of Pooled DNA Samples (Pool-Seq) Uncovers Complex Dynamics of Transposable Element Insertions in Drosophila melanogaster
Source: PLoS Genet. 2012 Jan 26;8(1):e1002487. doi: 10.1371/journal.pgen.1002487 (PMC3266889; doi:10.1371/journal.pgen.1002487)

2L

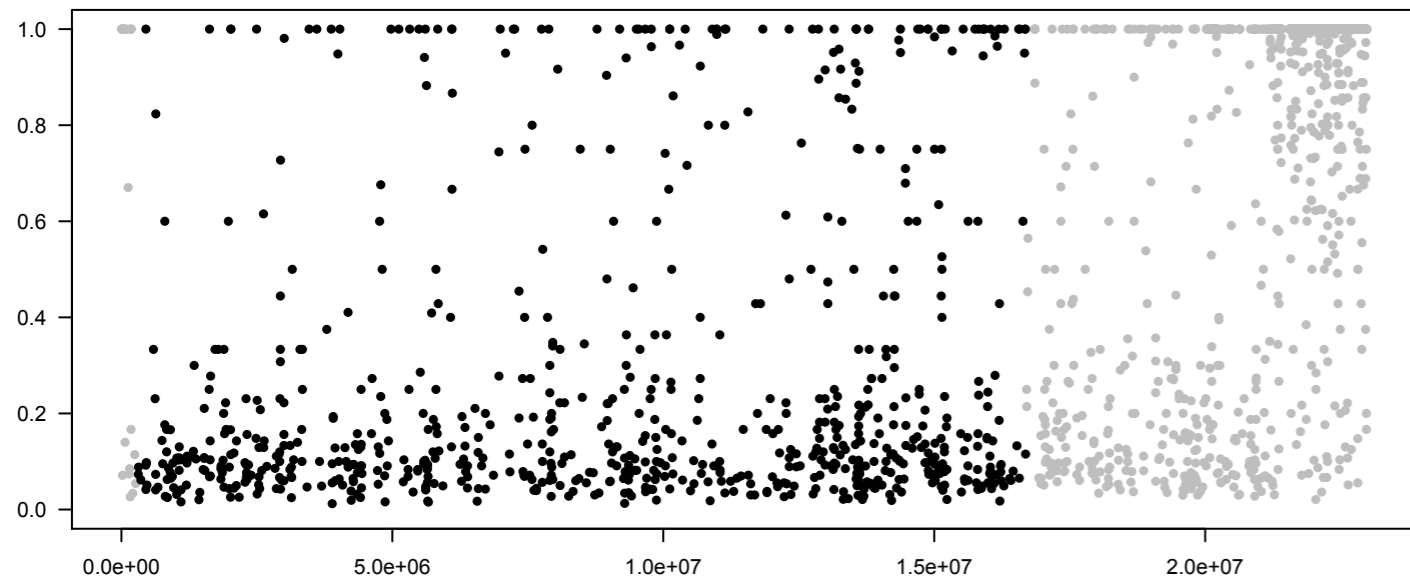

2R

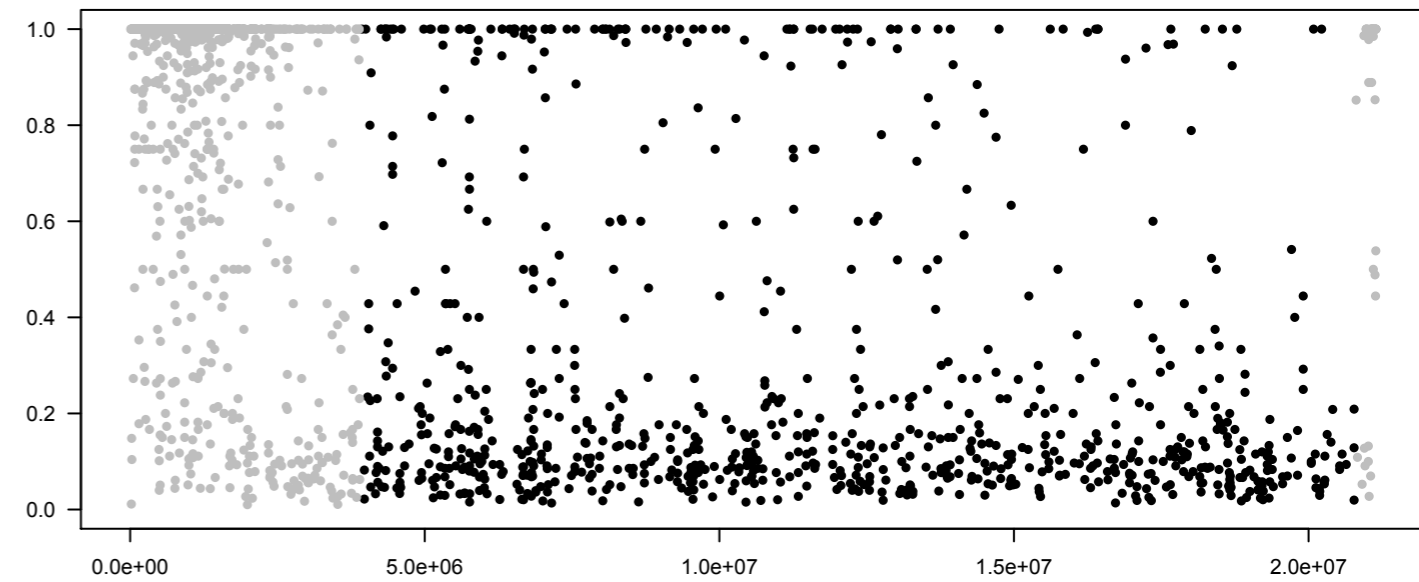

3L

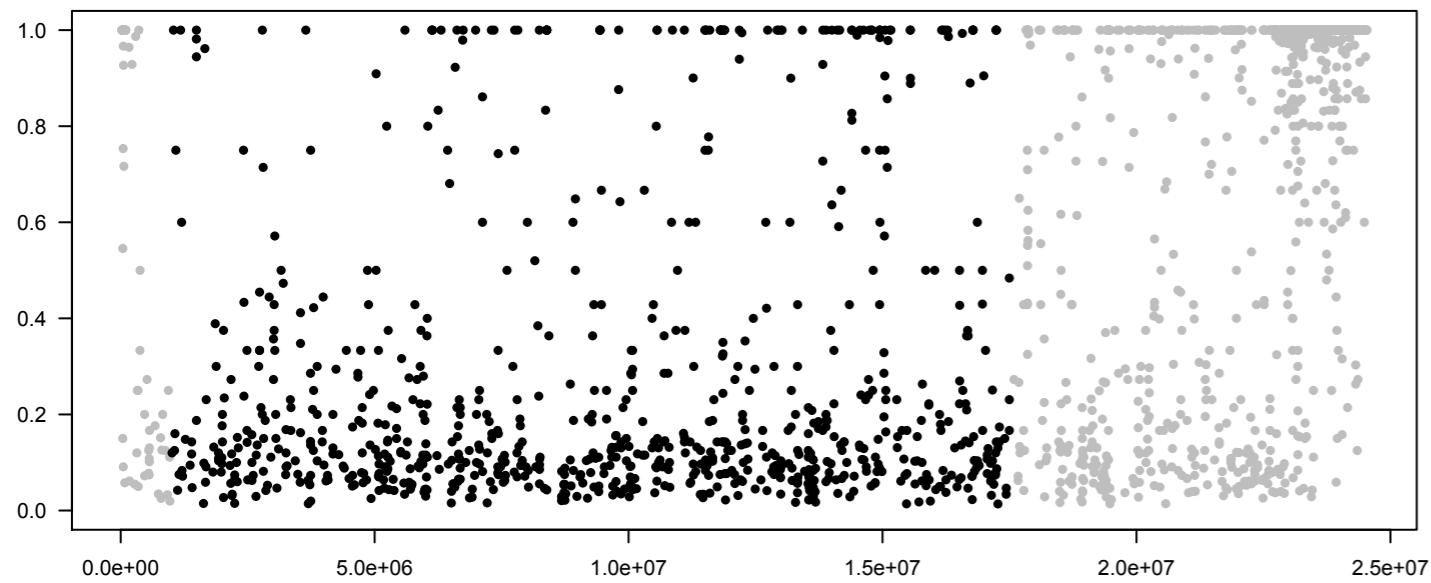

3R

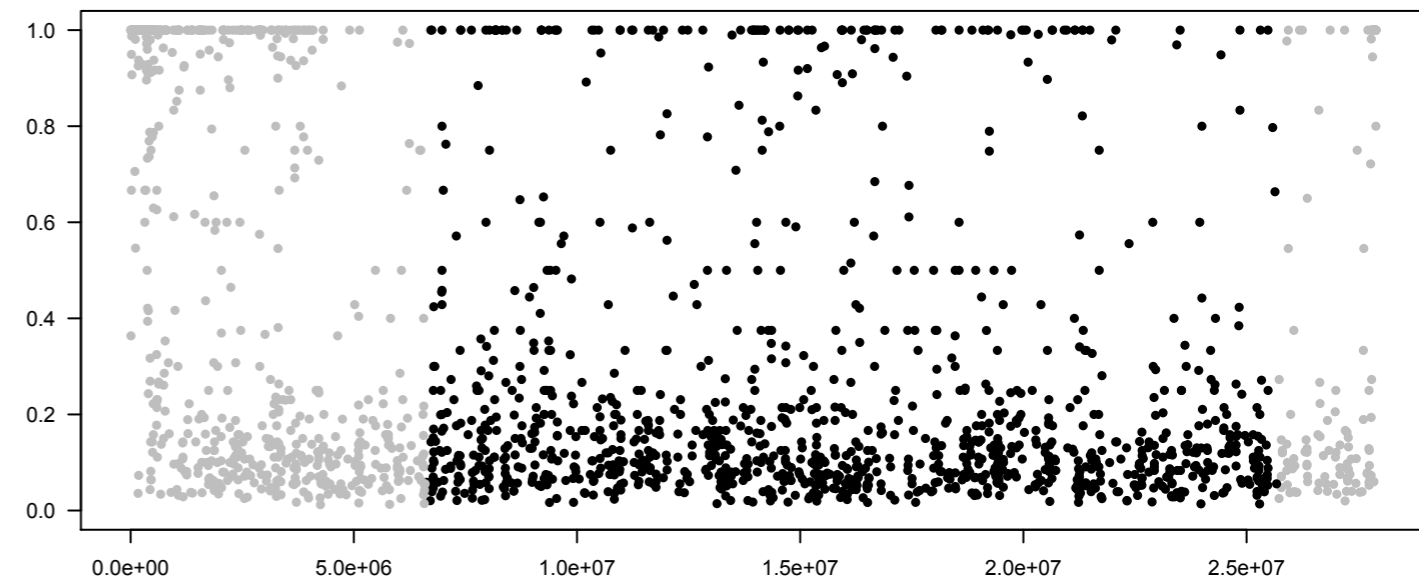

X

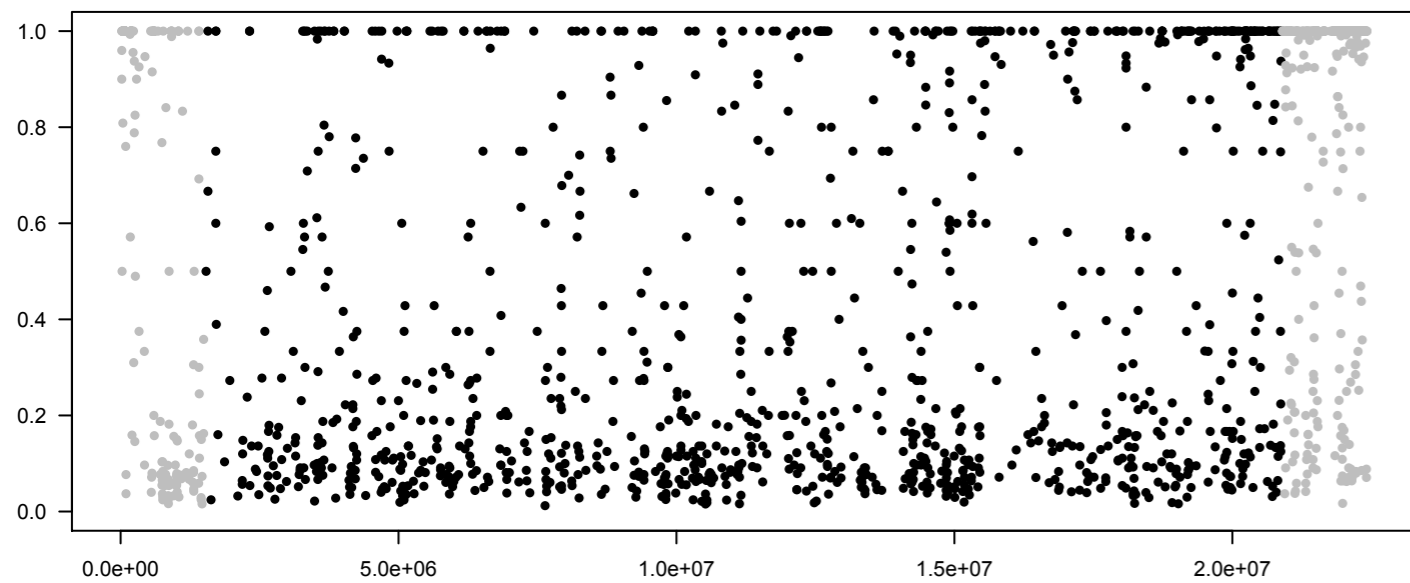

4

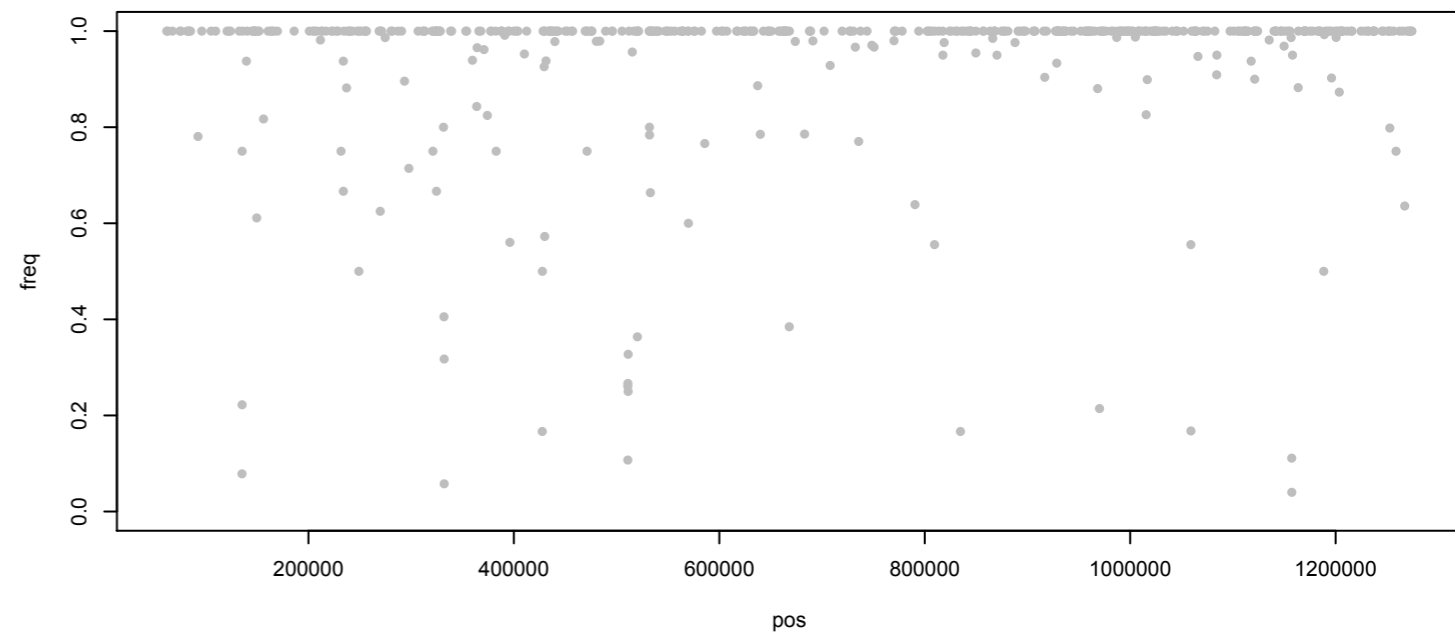

Supplement: Figure S1 — Distribution of TE insertions and their population frequencies in D. melanogaster. The x-axis shows the position in the chromosome and y-axis the population frequency of a TE insertion. Light grey insertions indicate low recombining regions (<1 cM/Mbp). (PDF) [file pgen.1002487.s001.pdf]

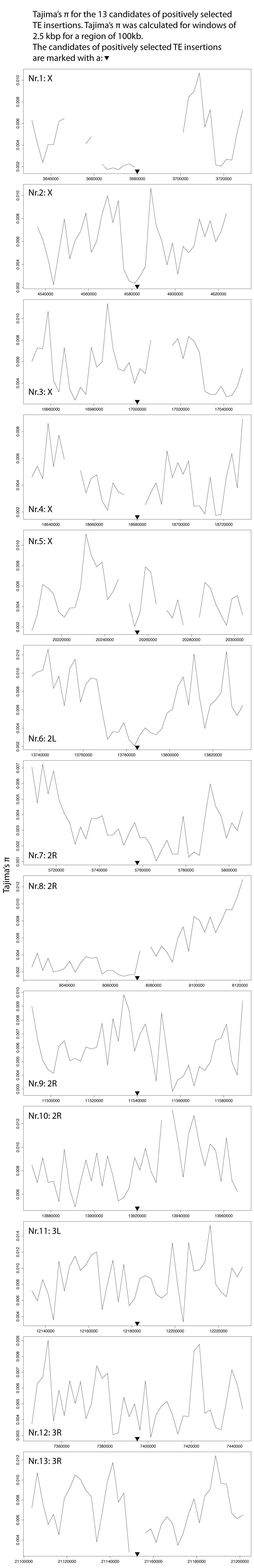

Supplement: Figure S2 — Nucleotide diversity in the vicinity of the 13 candidates of positively selected TE insertions. Nucleotide diversity was calculated for non-overlapping windows of size 2.5 kbp in a region of 100 kbp. (PNG) [file pgen.1002487.s002.png]
