# Supplementary material for: Sequencing of Pooled DNA Samples (Pool-Seq) Uncovers Complex Dynamics of Transposable Element Insertions in Drosophila melanogaster
Source: PLoS Genet. 2012 Jan 26;8(1):e1002487. doi: 10.1371/journal.pgen.1002487 (PMC3266889; doi:10.1371/journal.pgen.1002487)
Supplement: Text S1 — Supplementary discussion. (DOC) [file pgen.1002487.s011.doc]

# Discussion of the method:

**Strategies to deal with sequence divergence of TE insertions** For the protocol introduced in this work we map PE reads to a combined reference sequence, consisting of the repeat masked reference genome and the TE sequences used for repeat masking. There may be a considerable sequence divergence between individual TE insertions of the same family. We aim to mitigate this problem by using a redundant TE sequence database that contains several slightly divergent sequence entries for every TE family. However, this solution poses a new problem, as extensive sequence similarity between TE inserts of the same family will frequently lead to ambiguously mapping of reads. For example, the TE database of *D. melanogaster* (5.31) contains 2,153 entries for INE-1. We address this problem by considering TEs in a hierarchical fashion. If, for example, several mates of TE insertion fragments have been mapped to different entries in the TE sequence database, but all those entries refer to the INE-1 family, we can conclude that an INE-1 element has been inserted. In case the applied hierarchy level proves to be insufficient in reducing ambiguity, higher levels in the TE hierarchy may be used (e.g.: superfamily, order, class). Since the TE hierarchy is still debated [1,2,3], we have written our software such that the user can define the TE hierarchy. An alternative strategy, which we also employ, is to use liberal parameters for aligning the PE reads. In the present work we use BWA SW [4], which allows for mismatches and indels [5] and for only partial alignments of reads [5]. This is especially useful for our work, as for reads spanning a TE insertion, the alignment may be terminated at the TE insertion site.

**PE fragments aligning with different reference contigs** For 59.8 mil. of the PE fragments, both reads were aligned to the same contig, with 57.8 mil. mapping to the same reference chromosomes and 2.4 mil. to the same TE. For 1.4 mil. PE fragments, the two reads mapped to different reference chromosomes. For the majority of these PE fragments, one read aligns to an unassembled reference chromosome (Uextra:77%, U:7%). Thus future improvements in the reference assembly will likely reduce the number of PE fragments where the two reads map to different reference chromosomes.

**False positive TE insertion fragments** There are several issues that might lead to false positive inserts, or identification of an insertion that is not actually present. Problems during sample preparation may yield chimeric PE-fragments, in which the individual reads of a PE-fragment map to disjoint positions on different chromosomes, resulting in PE-fragments that incorrectly support the insertion of a TE. Inaccuracies in mapping may also result in PE-fragments incorrectly supporting the insertion of a TE. To reduce the impact of these problems, we required a minimum count of three paired end fragments supporting a TE insertion. We also required a minimum mapping quality of 15 for reads mapping to the reference genome, which should eliminate the majority of wrongly mapped reads (for details see [4]). Note that it is not desirable to require a minimum mapping quality for reads mapping to a TE, as the TE sequence database is highly redundant (see above), which will result in many ambiguously mapped reads having a mapping quality of zero.

**Orphan forward and reverse insertions** A TE insertion may be identified by PE fragments spanning the TE insertion from the 5’ direction and from the 3’ direction, which we denote as forward and reverse reads respectively (Figure 1A in the manuscript.) While it is expected that a given TE insertion is identified by forward and reverse reads, we frequently identified TE insertions only based on forward or reverse reads (Figure 1B in the manuscript). We propose the following explanations for this observation. First, these might be clustered or nested TEs insertions (about 21% of the TE insertions in *D. melanogaster* [6]). Since only TE insertions that can be anchored within a reference chromosome by PE fragments can be identified by our method, for clustered TE insertion frequently only a single insertion site will be detected. Second, they might result from imprecise repeat masking. In several cases, we noticed that the masked genomic region extends beyond the actual TE sequence, preventing mapping of reads to the masked region and causing a insertion site to be missed. Finally, many TE insertions segregate at low population frequencies. Due to sampling effects, only the forward or reverse insertions may be identified for those TE insertions. Despite these potential problems, we identified insertions from both forward and reverse reads for 3,030 (29.6%) of the TE insertions. From those 7,178 TE insertions that have only been identified by forward or reverse reads, 3,427 (47.7%) have a population frequency smaller than 0.2. Thus, sampling effects may to a large extent be responsible for not identifying TE insertion by both forward and reverse reads.

**Estimating the amount of false absence reads** Our approach of defining “presence” and “absence” reads, may yield a small fraction of false absence fragments (Figure 1C in the manuscript), leading to an underestimate of the true population frequency. We estimate the extent of this bias as follows. False absence reads are mostly due to small PE fragments (those where the reads are separated by fewer bases than expected; see also Figure 1C in the manuscript). In this analysis, false absence reads will only occur if the inner distance between paired reads is smaller than 26 bp (100 bp maximum range - 74 bp read length). We estimated the proportion of these reads using the distribution of distances obtained with BWA ALN version 0.5.7 [7]. Assuming a normally distributed inner distance [N(*μ* = 78.66, *σ^2^* = 16.99)], we expect about 0.1% false absence reads (inner distance <26bp). Note that overlapping PE reads (negative inner distance) are excluded from the analysis. We also obtained an empirical estimate of the fraction of false absence reads, using the insertions from *INE-1* families. *INE-1* has not been active for >3 million years [8,9], and many *INE-1* insertions were fixed prior to the split of the *D. melanogaster* and the *D. simulans* lineages [10]. Assuming that the 1,799 *INE-1* insertions found in the reference genome are invariably fixed, we estimate an average population frequency for these insertions of 97%. As some of these insertions may not actually be fixed, this 3% represents an upper limit to the false absence rate.

**Reproducibility of population frequency estimates** We assessed the reproducibility of our frequency estimates by comparing frequency estimates for the same TE insertion from forward and from reverse reads (Figure 1). For the 2,035 TE insertions identified by both forward and reverse reads, population frequency estimates obtained with corresponding pairs of forward and reverse insertions should be very similar, as they refer to the same TE insertion (Figure 1B in the manuscript). We found that the estimates were, indeed, highly correlated (Spearman’s rank correlation; P < 2.2e-16; rho = 0.902; Figure 1), demonstrating that these estimates are highly reproducible.


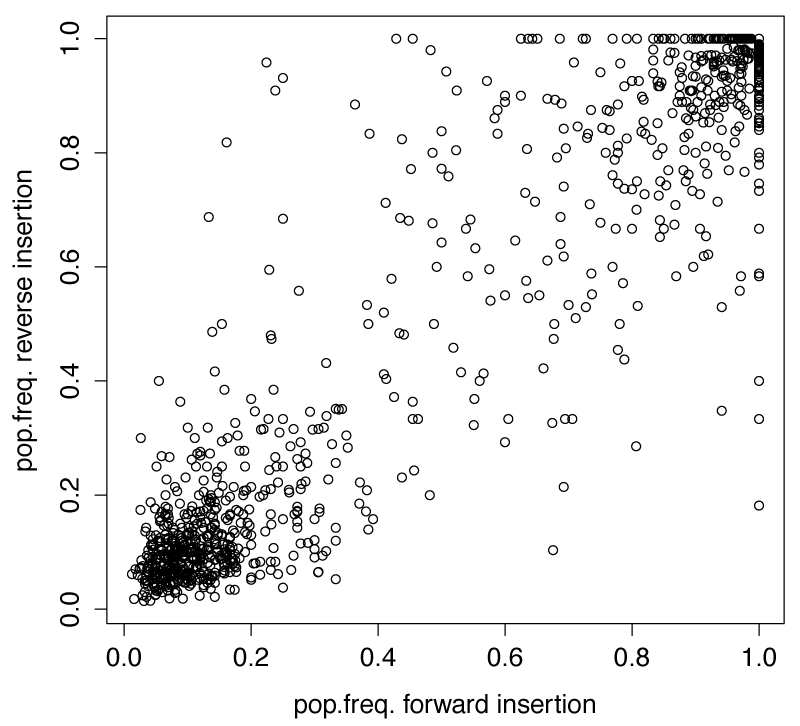


**Figure 1.** Population frequency of TE insertions as estimated by corresponding forward and reverse reads.

**Power to detect low frequency TE insertions** We also assessed the ability of our method to detect low-frequency insertions. The minimum population frequency that can be identified is difficult to assess genome-wide, as the coverage of insertion sites fluctuates. Although we found some TE insertions with population frequencies as low as 1%, the distribution of low population frequencies peaks around 6-7% (Figure 2). As a rough guideline, we estimate that we identify the majority of the TE insertions segregating at about 7%. It is possible to lower the threshold by decreasing the number of required PE-fragments that confirm the presence of a TE insertion, but this would also increase the number of false positive TE insertions.


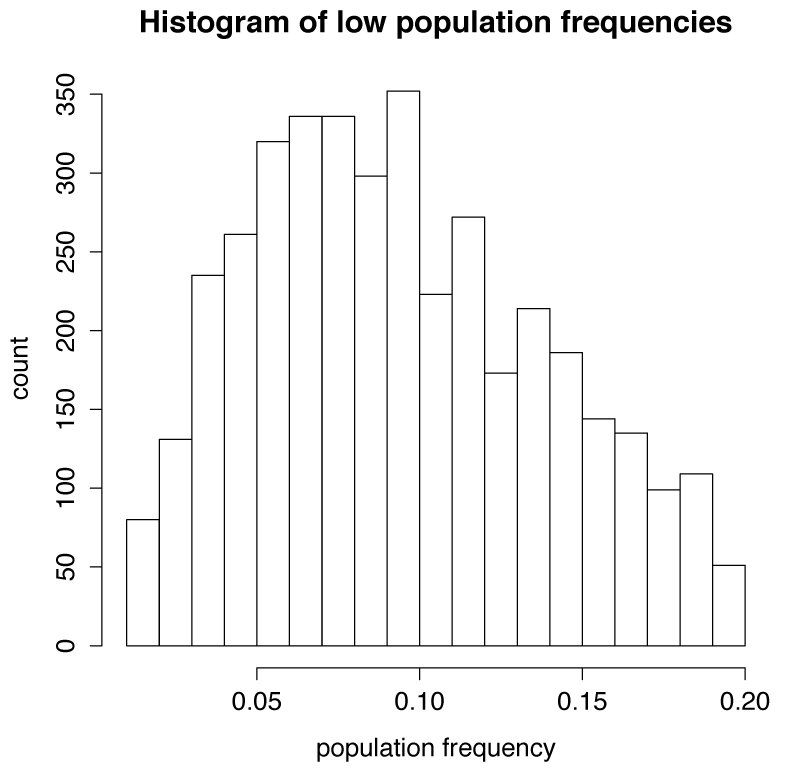


**Figure 2**. Histogram of TE insertions having low population frequencies

## Literature:

## 1. Kapitonov VV, Jurka J (2008) A universal classification of eukaryotic transposable elements implemented in Repbase. Nat Rev Genet 9: 411-412; author reply 414.

## 2. Seberg O, Petersen G (2009) A unified classification system for eukaryotic transposable elements should reflect their phylogeny. Nat Rev Genet 10: 276.

## 3. Wicker T, Sabot F, Hua-Van A, Bennetzen JL, Capy P, et al. (2007) A unified classification system for eukaryotic transposable elements. Nat Rev Genet 8: 973-982.

## 4. Li H, Durbin R (2010) Fast and accurate long-read alignment with Burrows-Wheeler transform. Bioinformatics 26: 589-595.

## 5. Smith TF, Waterman MS (1981) Identification of common molecular subsequences. J Mol Biol 147: 195-197.

## 6. Kaminker JS, Bergman CM, Kronmiller B, Carlson J, Svirskas R, et al. (2002) The transposable elements of the Drosophila melanogaster euchromatin: a genomics perspective. Genome Biol 3: RESEARCH0084.

## 7. Li H, Durbin R (2009) Fast and accurate short read alignment with Burrows-Wheeler transform. Bioinformatics 25: 1754-1760.

## 8. Singh ND, Petrov DA (2004) Rapid sequence turnover at an intergenic locus in Drosophila. Mol Biol Evol 21: 670-680.

## 9. Kapitonov VV, Jurka J (2003) Molecular paleontology of transposable elements in the Drosophila melanogaster genome. Proc Natl Acad Sci U S A 100: 6569-6574.

## 10. Wang J, Keightley PD, Halligan DL (2007) Effect of divergence time and recombination rate on molecular evolution of Drosophila INE-1 transposable elements and other candidates for neutrally evolving sites. J Mol Evol 65: 627-639.
